
JOURNAL INFORMATION
==============================
NLM Title Abbreviation: Med Chir Rev
NLM Journal ID: 0034617

The Medico-Chirurgical Review

Publisher: Biomedical Journal Digitization Project

ARTICLE INFORMATION
==============================
PMCID: PMC4966195
PMID: 29917617
Article version: 1
Subjects: Reviews

On Functional Disorders of the Heart

Print publication date: 1828 Jul 1
Volume: 9
Issue: 17
First page: 20
Last page: 24
1828 Apr 12
License: This work is free of known copyright restrictions. For more information, please see PMC Back Issue Digitization.
License URL: https://creativecommons.org/publicdomain/mark/1.0/

==============================

The reader will find recommended in Dr. Burne's book a well-timed use of aperients and enemata, an attentive observation of the colour and quality of the discharges—a strict attention to diet—a meeting of indications as they arise, not upon any insulated or empirical principles, but upon the broad basis of the views that he has taken of the nature and seat of the disease. In short, we consider this book as affording a good example of the superior prudence and efficiency of "a mixed treatment," guided by observation, and based upon a rational humoral pathology, as compared with the generalizing compendious practice of intolerant localists. The work before us, though a very useful addition, as it stands, to our stock of practical knowledge on the subject of typhus, would be at least a more agreeable one, if its pages were occasionally relieved by a little shading from either ancient, modern, or cotemporary authorities. Had Dr. B. not disdained to read, or, having read, to quote, he would not have omitted to notice the eruptive disease* peculiar to the adynamic patient, between the second and the seventh day—the anatomical characters of *petechiæ* as differing from the typhoid exanthem†—the tendency to various kinds of natural crisis, &c. We conceive it to be our duty to deprecate this autocratic, exclusive plan of writing upon a subject, which has been so ably investigated by so many great men—this setting us down to a solitary meal, however nutritious, when so many instructive guests might have been invited to meet us. If an author be either too lazy, or too proud, to strengthen his own conclusions, by an opposition to, or a comparison with, those of others; his readers will try to invalidate them by this very process, and will often succeed. To him who reads, all written authorities are of equal weight, except where the author is personally known. Notwithstanding these errors of omission, we can safely recommend Dr. Burne's book to the favourable notice of such of our readers, as may be disposed to take a plain, common sense, useful view of continued fever.

The late period at which Dr. Burne's work appeared (after the construction of this article)—the hurried glance which we have been obliged to take of it—and other considerations unnecessary to be stated—leave us the option (and we shall shortly have the opportunity) of again reverting to certain doctrines and opinions in the publication, when noticing the section on fever in Dr. Bright's work, of which one more analytical article yet remains for insertion in the course of the present number.

---

### II.

### *On Functional Affections of the Heart and Arteries.* M. LAENNEC.

IN our last number we noticed the subject of neuralgia of the heart, and also angina pectoris. We shall now advert to some other nervous affections of this organ.

#### PALPITATION.

Every beating of the heart which is sensible and unpleasant to the individual, and, at the same time, more frequent than natural, is termed palpitation. The

* Vide Thucidides, Raikem, and Bianchi, Boin, Pinel, &c.
† Rouchoux.

pulsation is often audible by the patient, and even by the byestander. If a person lies in a horizontal posture, during palpitation of the heart, he will hear, in that ear which is next the pillow, a pulsation double in number to that at the wrist. This arises from his hearing the alternate contractions of the auricles and ventricles. In many cases, there is only an increase of frequency in the actions of the heart, while the patient imagines, from his sensations, that there is also great increase of force. Laennec has known this kind of palpitation go on for eight days, the pulse remaining extremely small and weak, and from 160 to 180 in the minute. In healthy persons, and from the excitement of moral or physical causes, there will be an increase both of force and frequency in the heart's action. As the sound and sphere of the heart's pulsations are much increased during palpitation, we should never draw any conclusions from auscultation at such times. We must wait till the heart is in its usual rate of going.

But to speak of purely nervous palpitation, unaccompanied by any organic change in the heart, it may be observed that this kind is often much more troublesome and distressing to the individual than that which is dangerous from its cause. Far from being removed by the most complete repose, it is, in general, most distressing during the early part of the night. It often prevents sleep—and it is often removed, or at least mitigated, by moderate exercise. There is no complaint which is more liable to lead medical men, especially young practitioners, astray, than palpitation of the heart. The following passage from Laennec is in perfect accordance with our own observations.

"The purely nervous palpitations consist in an increase of the impulse, sound, and particularly of the frequency of the heart's pulsations. A feeling of internal agitation, particularly in the head and abdomen, always accompanies them; also a limpid watery condition of the urine. The duration of palpitations of this kind is very variable: they may be momentarily excited by mental emotion; while, at other times, they seem to originate without any obvious cause, and continue for several years, especially in young persons who are at the same time both nervous and plethoric.—It is commonly imagined that such an habitual over-action of the heart as such palpitations imply, must at length give rise to hypertrophy of this organ. This is possible; but I must say that I have never seen any proof of the accuracy of this opinion. On the other hand, I am acquainted with individuals who have been habitually subject to affections of this kind, and who nevertheless exhibit no positive sign either of hypertrophy or dilatation."

The physical signs (as revealed by examination with the ear) which distinguish nervous palpitation from that dependent on organic disease, are thus characterised by Laennec.

"In nervous palpitation, the first impression conveyed by the stethoscope is that the heart is not enlarged. The sound, though clear, is not heard loudly over a great extent of chest; and the impulse, although appearing considerable at first, is really not great, as it never sensibly elevates the head of the observer. This last sign seems to me the most important and certain of any, when taken in conjunction with the frequency of the pulsations. These are always quicker than natural,—being, most frequently, from eighty-four to ninety-six in the minute. Nervous palpitations are rarely accompanied by any sign of determination of blood to the head or chest, except in old persons."

Palpitation of the heart is often increased by the very means which are taken to subdue it. Dr. Parry made it fashionable to attribute all nervous diseases to increased fulness or impetus of the blood-vessels, and therefore the lancet was freely used in palpitations. There can be no doubt that there are many sedentary females who eat a great deal too much, and walk a great deal too little, and who, consequently, have a plenitude of the vascular system, and a preternatural mobility of nerve. These, when affected with palpitation, are benefited by blood-letting, abstinence, and even purgation. But, in most other subjects, and especially in hypochondriacal and hysterical individuals, the depletory practice increases the palpitation. The complaint is, in fact, extremely indomitable, and it is a great object to be able to say that it *is* nervous palpitation, and unaccompanied by danger. When a patient is thoroughly satisfied on this point, he or she will be less anxious about a cure, and more easily reconciled to the presence of an uncomfortable companion. There are, however, some means which occasionally relieve or remove this complaint. As this affection seldom exists, without some cause which can be traced, by accurate investigation, to errors in regimen, mental distress, or derangement of some corporeal function, so this inquiry should never be omitted. At all events, there can be no safer or more effectual plan of treatment than that which is based on temperance, regularity, and the improvement of any deranged function. Failing in these efforts, we may prescribe a steady system of exercise—the shower-bath—and certain *sedatives*, in which we have so great faith. The mobility of the nervous system is best reduced by that which gives natural tone and strength to the whole system. Plain food, regular exercise, and early hours, will do more than all the assafœtida, bark, and valerian, in Apothecaries' Hall.

### Irregularities of Action.

These may exist without palpitation. Irregularity of this kind is often met with in elderly people, without any disturbance of the general health. Sometimes, amid a series of pulsations, very unequal among themselves, a single one will occur one half shorter than the rest. This produces something like an intermission—and it completely resembles the latter if the pulsation be very weak. "These irregularities, as to frequency, take place most usually in persons affected with dilatation of the heart." The variations under consideration occur only in the heart, as heard by the ear or stethoscope—they make no sensible impression on the pulse, as felt at the wrist. We shall, therefore, pass on to more palpable irregularities of the heart's action.

### Intermissions of Pulsation.

By this we understand, of course, a sudden and momentary suspension of the pulse, during which the artery is no longer perceptible beneath the finger. The duration of this intermission is very variable—being sometimes longer, sometimes shorter, than a common pulsation. There are two kinds of intermission—one *real*, consisting in an actual suspension of the heart's contraction—the other *false*, resulting from contraction so feeble as to be incapable of perception by the finger on the artery. Intermissions of the first kind are most common—they are frequent in old age, even when the health is good—or when the indisposition is only slight. "In middle age, they are *only* observed in certain diseased states of the heart, particularly hypertrophy of the ventricles, and during palpitation." In this, we cannot agree with Laennec. We have seen so many instances, "in middle age," where temporary intermissions of the pulse were occasioned by flatulence, indigestion, acidity in the stomach—nay even by emptiness of that organ,—that we rarely attribute any importance to this phenomenon, unless it be accompanied by other symptoms indicative of disease in the central organ of the circulation. Our author informs us that, by means of the stethoscope, we can clearly ascertain that "this species of intermission always succeeds the contraction of the auricles—it therefore differs only from the natural quiescence after this contraction in the irregularities of its recurrence." "If," says he, "in our examinations, we content ourselves with feeling the pulse, without applying the stethoscope, we shall, of necessity, confound this true intermission with the false one formerly mentioned produced by variations in the duration and force of the heart's pulsations."

"The last species of intermission is that which consists in the absence of one complete pulsation, recurring sometimes with an exact periodicity, after longer or shorter intervals, the pulse being in other respects regular. This pulse constitutes, according to Solano, the precursor of a critical diarrhœa. This peculiarity of the circulation is by no means rare; I have observed it frequently in some epidemics, but not at all in others, owing no doubt to the particular constitution that prevailed. This kind of intermission corresponds more frequently to a contraction of the ventricles, much weaker than the rest, than to a real interruption of their action; and, indeed, in such cases we often perceive an extremely feeble pulsation in place of a total intermission."

In a note appended to this passage, Dr. Forbes states as follows:

"In certain cases of diseased heart I have observed this species of intermission under a form which was sometimes productive of curious results. Every second pulsation was so feeble as to be altogether or almost entirely imperceptible. In the former case, the pulse appeared to be quite regular and slow; but, while in the act of feeling it, the intermediate or *latent* pulsation (if I may use the expression) became suddenly distinct, and the pulse was instantly *doubled*. In this manner I have known the same patient with a regular pulse at fifty or sixty, and a regular pulse at one hundred or one hundred and twenty, within the space of three minutes."—*Transl.*

Dr. Johnson was the first to point out this species of intermission several years ago, in the case of a gentleman residing in Bond-street, a patient under the care of Mr. Cosgreave of this metropolis. In this case, the ventricular actions were usually double those of the tangible arteries. But when any feverish excitement took place, the pulse became double the usual number or more, at the wrist, and corresponded exactly with the pulsations of the heart against the ribs. In by far the greater number of cases where we have observed intermissions of the pulse, there was an action—evidently a ventricular action of the heart, at the moment of the intermission at the wrist. Laennec has filled some pages with very obscure, not to say unintelligible reasoning respecting certain peculiarities of the pulse, without attempting any explanation of the cause of intermissions. One of the conclusions to which this great pathologist has come, is, in our opinion, a great error—namely, an independent pulsation in the arteries, without any impulse from the heart.

From an attentive observation of this phenomenon (intermission of the pulse) and where we have had very good opportunities of investigation, we have come to the conclusion that, in all cases, it depends on an unsuccessful action or contraction of the ventricle—not on an intermission of the ventricular contraction. The causes, however, of this abortive action of the ventricle are various. In very many cases, it is dependent on sympathetic associations of the heart with other organs, especially with the abdominal viscera; in which cases, the intermission of the pulse is not constant, but only temporary. Where there is a permanent irregularity in the action of the heart or in the pulse, we believe there is generally some valvular disease, or alteration of structure. It may admit of much doubt, indeed, whether disease of the semilunar valves, or of the mitral valve be most productive of intermissions of the pulse. For our own parts, we are inclined to impute permanent intermissions more to imperfections in the former than in the latter apparatus. It is curious that neither Laennec nor his translator has alluded (as far as we can see) to valvular disease, as the cause of intermissions and other irregularities (we always mean *permanent* irregularities) of the pulse. Yet we think these irregularities, when not merely temporary nervous affections, are, in three cases out of four, dependent on this cause.

SPASM OF THE HEART—WITH BELLOWS-SOUND AND PURRING-THRILL.

Although the sounds above-mentioned frequently attend organic diseases of the heart, yet it is certain that they may exist in consequence of a purely nervous affection. But in these cases, " it is always attended by symptoms which constitute a real state of disease." The bellows-sound is most commonly heard in hypochondriacs—particularly in those of a sanguine and plethoric temperament—in which cases the sound is usually heard in some of the arteries at the same time—frequently passing from one to the other. It is sometimes continuous—sometimes intermittent:—in the latter case, it recurs on the slightest agitation of body or mind. The symptoms which accompany it are the more severe in proportion as the sound is greater, more continuous, and extending to a greater number of arteries.

" When it is very constant and distinct, but confined to the heart, there is almost always more or less dyspnœa, with a feeling of greater or less debility, so that the patient can, in many cases, hardly walk. These symptoms are still more marked, if the purring-thrill accompanies the bellows-sound. There is commonly but slight nervous agitation, particularly when the patient is quiet; but on attempting to walk rather quick, or for any length of time, he is soon out of breath, and, in the severer cases, the head becomes confused."

When this affection is unconnected with any organic lesion of the heart, it must be treated as a nervous complaint.

NEURALGIA OF THE ARTERIES.

Pains more or less acute, continued, or intermittent, sometimes follow the course of the arteries, and appear to have their seat in the nervous filaments supplied to these vessels by the ganglionic centre. They occur most frequently in hypochondriacs and in nervous females. A blister to the part thus affected, is considered by Laennec as the most effectual application.

PRETERNATURAL PULSATIONS OF ARTERIES.

These are regarded by Laennec as convincing proofs " that the arteries have an action of their own, independent of that of the heart." When any one will shew us a pulsation in an artery, when there is no corresponding ventricular contraction in the heart, then, and not till then, will we believe that the arteries can pulsate independently of any impulse from the heart. Such a phenomenon we have never yet seen—and we verily believe that such a phenomenon has never been seen or felt by man. But Laennec says that one carotid will be found to beat more strongly than another—and this is another proof of the dependence in question. We do not think so. If the arteries have a *pulse* independent of the heart, that pulse must consist in the *distention* of the vessel—for its contraction surely could not be felt by the finger. And is Laennec or any other man prepared to say that the arteries have a power of *self-distention* sufficient to constitute a pulse, or throb, independent of the rush of blood sent from the heart? If one artery is found to beat more strongly than another, are we sure that there is nothing which impedes the flow of blood *into* the latter, or *out of* the former? We apprehend that the inequalities of pulsation in arteries are more dependent on such circumstances than on any power which an artery may possess of more strongly dilating at one time or place than at another. Laennec instances the increased pulsation of an artery leading to an inflamed part, as to a whitlow. Is not this a case directly in point, and corroborating our position?
